# Comparative outcomes of middle meningeal artery embolization, burr hole drainage, and neuroendoscopic-assisted evacuation with MMA disconnection for chronic subdural haematoma: a multicenter retrospective cohort study

**DOI:** 10.3389/fneur.2026.1975364

**Published:** 2026-10-19

**Authors:** Xudong Song, Yuan Zhang, Bo Luo, Xiyuan Tang

**Affiliations:** 1 Department of Neurosurgery, Capital Medical University Affiliated Beijing Anzhen Hospital Nanchong Hospital, Nanchong, Sichuan, China; 2 Department of Neurosurgery, Guangyuan Central Hospital, Guangyuan, Sichuan, China

**Keywords:** burr hole drainage, chronic subdural haematoma, middle meningeal artery disconnection, middle meningeal artery embolization, neuroendoscope

## Abstract

**Objective:**

To compare the efficacy, safety, and resource utilization of middle meningeal artery embolization (MMAE), burr hole drainage (BHD), and neuroendoscopic-assisted evacuation with middle meningeal artery disconnection (NE-MMAD) for chronic subdural haematoma (CSDH).

**Methods:**

A retrospective analysis was performed on 265 patients with CSDH, divided into MMAE (*n* = 46), BHD (*n* = 152), and NE-MMAD (*n* = 67) groups. The primary outcome was the 6-month recurrence rate. Secondary outcomes included neurological improvement rate, reoperation rate, operation time, hospital stay, number of imaging follow-ups, and complications.

**Results:**

The recurrence rates for MMAE and NE-MMAD (4.35 and 4.48%, respectively) were significantly lower than that for BHD (19.74%, *p* = 0.001). The neurological improvement rate was highest in the NE-MMAD group (88.06%, *p* = 0.003). The reoperation rates for MMAE and NE-MMAD were both lower than that for BHD (*p* = 0.002). In the MMAE group, 5 patients (10.87%) underwent salvage BHD due to delayed resolution of mass effect. MMAE had the shortest operation time (31 min) and hospital stay (7 days), while NE-MMAD had the longest operation time (70 min); both MMAE and NE-MMAD required fewer imaging follow-ups than BHD (all *p* < 0.05). No significant difference in complication rates was observed among the three groups; however, one patient in the MMAE group experienced permanent blindness due to inadvertent embolization through a dangerous anastomosis.

**Conclusion:**

In this cohort, MMAE and NE-MMAD were associated with lower recurrence compared with BHD, and NE-MMAD was associated with the greatest neurological improvement. BHD may be preferred for first-onset, non-septated haematomas; MMAE may be preferred for older patients or those with coagulopathy, but the risk of blindness warrants caution; NE-MMAD may be preferred for septated, organized, recurrent, and markedly space-occupying haematomas. Clinical decision-making should integrate haematoma characteristics, patient status, and available medical resources to guide individualized stratified treatment.

## 1 Introduction

Chronic subdural haematoma (CSDH) is one of the most common neurosurgical cranial conditions, predominantly affecting the elderly population. Its incidence has been rising annually due to population aging and the widespread use of anticoagulant and antiplatelet agents (1, 2). CSDH has an insidious onset, with nonspecific clinical manifestations that are easily confused with degenerative conditions such as brain atrophy and Alzheimer’s disease (3).

The traditional pathophysiological mechanism of CSDH was once attributed to slow venous bleeding from torn bridging veins (4). However, with advancing research, CSDH is now recognized as an active disease driven by aseptic inflammation, characterized by pathological angiogenesis and hyperfibrinolysis (4). Following minor head trauma, the dural border cell layer triggers an inflammatory cascade, leading to the formation of inner and outer membranes rich in neocapillaries (5). The middle meningeal artery (MMA), as the main arterial supply to the dura mater, gives off branches that nourish the neovasculature of the haematoma’s outer membrane (6, 7). These newly formed vessels have an incomplete basement membrane and abnormally increased permeability, and under a locally hyperfibrinolytic microenvironment, they undergo recurrent microhaemorrhages, driving progressive haematoma expansion (8). Thus, CSDH is now viewed as a metabolically active angiogenic disease characterized by sustained inflammation, hyperfibrinolysis, and pathological neovascularization.

Currently, the treatment of CSDH has shifted from simple haematoma evacuation to a comprehensive model that combines evacuation with etiological intervention (9). Burr hole drainage (BHD) can rapidly relieve mass effect, but it cannot arrest the persistent leakage from neovessels in the outer membrane, resulting in a recurrence rate as high as 14–37% (10, 11). Middle meningeal artery embolization (MMAE) occludes the MMA trunk via an endovascular approach, cutting off the nutritional supply to the neovessels of the outer membrane at the source. Recent studies have confirmed that MMAE can reduce the recurrence rate to approximately 4% (12); however, this technique cannot immediately remove the established haematoma mass, and its clinical efficacy exhibits a “time-lag window” (13). Moreover, MMAE is highly dependent on interventional facilities and carries the rare but catastrophic risk of permanent blindness due to inadvertent embolization through dangerous meningo-ophthalmic anastomoses (14). In contrast, neuroendoscopic-assisted evacuation with middle meningeal artery disconnection (NE-MMAD) allows direct visualization of the haematoma cavity and simultaneous disconnection of the MMA. This technique offers unique advantages in septated, organized, and recurrent CSDH (15), with a reported postoperative recurrence rate as low as 3.8% (16), and does not require interventional equipment.

However, current evidence on the selection of surgical modalities for CSDH largely derives from single-arm cohorts or indirect comparisons between two groups; direct comparative studies involving all three techniques are still lacking. Based on multicenter retrospective data, this study systematically compares the clinical efficacy, safety, and healthcare resource utilization of MMAE, BHD, and NE-MMAD for CSDH, aiming to provide evidence-based support for individualized surgical decision-making.

## 2 Methods

### 2.1 Study design and population

This was a multicenter retrospective observational study. A total of 265 patients with CSDH who were hospitalized in the Department of Neurosurgery at Nanchong Central Hospital and Guangyuan Central Hospital between July 2020 and February 2026 were enrolled. The inclusion criteria were: (1) clinical and radiological diagnosis of CSDH with a symptom duration of ≥3 weeks; (2) age between 18 and 90 years; (3) presence of neurological symptoms (headache, limb weakness, gait disturbance, cognitive decline, etc.); (4) imaging evidence of a maximum haematoma thickness >10 mm or midline shift >5 mm; and (5) treatment with MMAE, BHD, or NE-MMAD with complete clinical data.

The exclusion criteria were: (1) acute or subacute subdural haematoma; (2) concurrent intracranial aneurysm, arteriovenous malformation, or intracranial tumor; (3) severe coagulation dysfunction (platelet count <70 × 10^9^/L or INR > 2.0); (4) pregnancy or lactation; (5) life expectancy <6 months due to other diseases; and (6) loss to follow-up or incomplete follow-up data.

The local selection criteria and participant flow are summarized in Supplementary Figure S1.

### 2.2 Group allocation and surgical procedures

Patients were divided into three groups according to the surgical modality: MMAE group (*n* = 46), BHD group (*n* = 152), and NE-MMAD group (*n* = 67).

#### 2.2.1 MMAE group (*n* = 46)

The procedure was performed under general or local anesthesia. A 5F femoral artery sheath was placed via femoral artery puncture. Initial internal and external carotid angiography was performed to delineate the course and territory of the MMA. Thereafter, a guiding catheter was positioned distal to the origin of the internal maxillary artery in the external carotid artery. Using roadmap guidance, a microcatheter was superselectively navigated into the MMA trunk, targeting both the anterior and posterior branches of the MMA. Onyx (ethylene-vinyl alcohol copolymer) was used as the embolic agent, which was slowly injected under fluoroscopic monitoring until the target vessels were no longer opacified (Figure 1).

**Figure 1:**
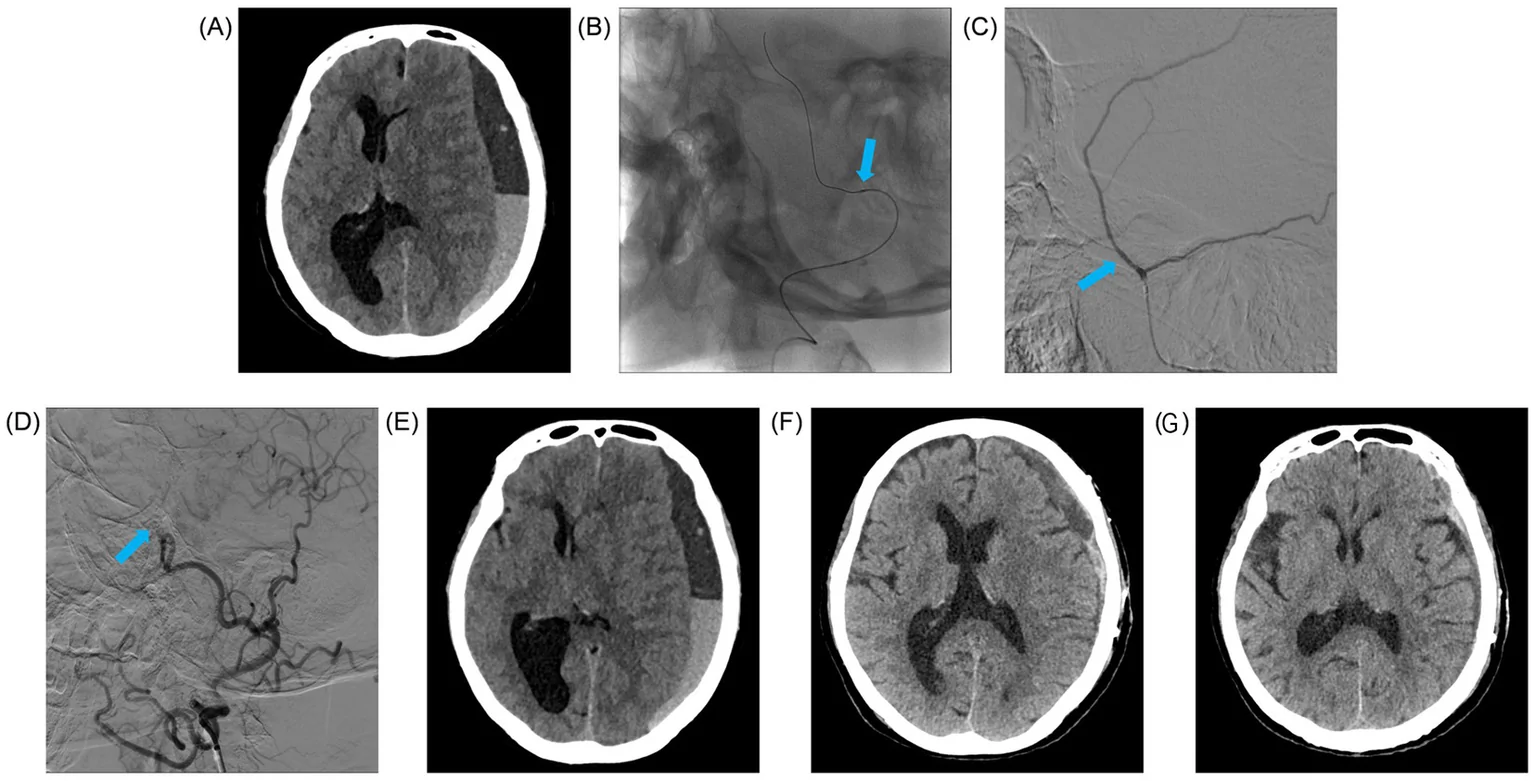
Intraoperative angiography and postoperative CT follow-up of MMAE for CSDH. **(A)** Preoperative CT; **(B)** intraoperative DSA showing the microcatheter superselectively advanced into the MMA trunk (blue arrow); **(C)** preoperative roadmap confirming the microcatheter tip position and the target vascular anatomy (blue arrow); **(D)** post-embolization angiography showing interruption of MMA supply (blue arrow); **(E)** CT on postoperative day 1; **(F)** CT at 1 month postoperatively; **(G)** CT at 6 months postoperatively.

#### 2.2.2 BHD group (*n* = 152)

The burr hole site was localized at the thickest portion of the haematoma based on preoperative cranial CT, usually near the parietal tubercle. After perforating the skull, a cruciate incision was made through the dura mater and the outer membrane of the haematoma, and the haematoma was slowly evacuated for decompression. The haematoma cavity was irrigated repeatedly with warm normal saline until the return fluid was clear. Two 10F silicone drainage tubes were then placed into the frontal and occipital aspects of the cavity, respectively; the distal ends of the tubes were tunneled subcutaneously and connected to closed drainage bags postoperatively. The drainage tubes were removed 2–3 days after surgery based on the drainage volume and follow-up CT findings (Figure 2).

**Figure 2:**
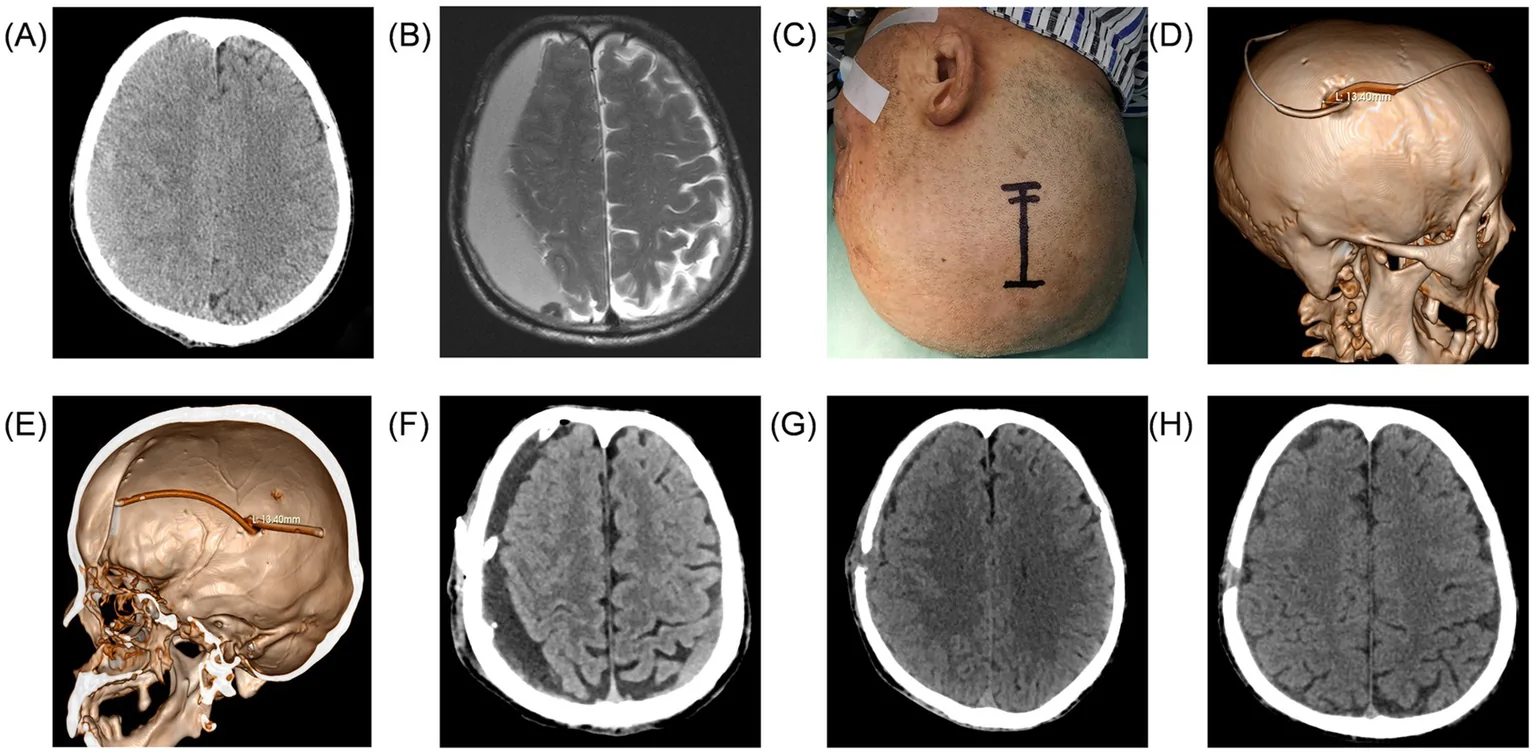
Surgical procedure and postoperative CT follow-up of BHD for CSDH. **(A)** Preoperative CT; **(B)** preoperative MRI; **(C)** scalp marking for burr hole drainage; **(D)** 3D reconstruction showing the drainage tube *in situ* (burr hole diameter: approximately 13.40 mm); **(E)** 3D reconstruction (lateral view) showing drainage tubes placed in the frontal and occipital aspects of the haematoma cavity, respectively; **(F)** CT on postoperative day 1; **(G)** CT at 1 month postoperatively; **(H)** CT at 6 months postoperatively.

#### 2.2.3 NE-MMAD group (*n* = 67)

Preoperatively, thin-slice CT data (in .dcm format) were imported into 3D Slicer software for three dimensional reconstruction to localize the groove of the MMA on the inner table of the skull and its surface projection, thereby marking the surgical incision (the center of- the bone flap was routinely positioned 1 cm posterior and superior to the pterion). The patient was placed in the supine position under general anesthesia with the head turned to the contralateral side. According to the preoperative plan, a straight incision approximately 4–5 cm in length was made in the temporal region, and the skin, subcutaneous tissue, and temporalis muscle were sequentially incised to expose the underlying bone. A craniotomy with a diameter of approximately 3–4 cm was performed using a high-speed drill, with the center of the bone window located at the surface projection of the frontal branch trunk of the MMA. After dural suspension, the frontal branch trunk and its branches of the MMA were exposed and disconnected using bipolar coagulation. The dura mater and the haematoma capsule were then incised in a cruciate fashion, and the haematoma was slowly evacuated. A 0° or 30° neuroendoscope (4 mm in diameter) was introduced to inspect the haematoma cavity under direct visualization. Liquid and solid haematoma components were removed with a suction device, and septations as well as active bleeding points were coagulated and severed. The cavity was repeatedly irrigated with warm normal saline until clear. A single drainage tube was placed in the frontal aspect of the haematoma cavity, and the bone flap was replaced and secured with connecting plates. The scalp was closed in layers. Postoperative drainage tube management was the same as that in the BHD group (Figure 3).

**Figure 3:**
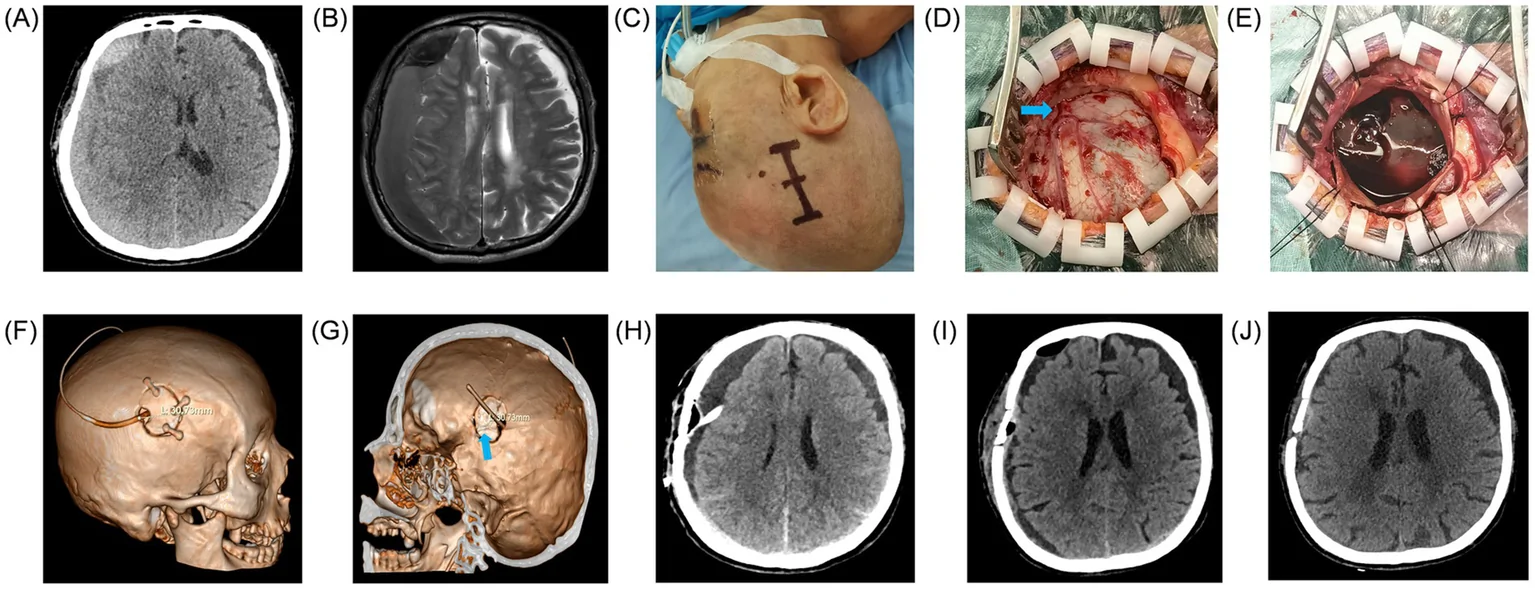
Surgical procedure and postoperative CT follow-up of NE‑MMAD for CSDH. **(A)** Preoperative CT; **(B)** preoperative MRI; **(C)** preoperative scalp marking for NE‑MMAD; **(D)** intraoperative view showing exposure of the main trunk and branches of the frontal branch of the MMA (blue arrow); **(E)** haematoma cavity after dural suspension; **(F)** 3D reconstruction showing the bone window (diameter approximately 30.73 mm) and a drainage tube placed in the frontal aspect of the haematoma cavity; **(G)** 3D reconstruction (lateral view) showing the bone window and the groove of the MMA (blue arrow); **(H)** CT on postoperative day 1; **(I)** CT at 1 month postoperatively; **(J)** CT at 6 months postoperatively.

### 2.3 Data collection and outcome measures

Baseline data were collected for all patients, including age, sex, haematoma side (unilateral/bilateral), maximum haematoma thickness, the presence of septated haematoma, degree of midline shift, admission modified Rankin Scale (mRS) score, use of anticoagulant/antiplatelet agents, and comorbidities (hypertension, diabetes mellitus, etc.). Septation was defined as the presence of internal membranes or multiloculated compartments within the haematoma on preoperative CT and/or MRI.

The primary outcome was the 6-month haematoma recurrence rate. Recurrence was defined as an increase in haematoma thickness on follow-up cranial CT, with or without worsening of clinical symptoms, and irrespective of whether repeat surgical intervention was required.

Secondary outcomes included: (1) neurological improvement rate, defined as a decrease of ≥1 point in the mRS score at follow-up compared with admission; (2) reoperation rate for haematoma recurrence; (3) any additional surgery, defined as reoperation for recurrence plus salvage burr hole drainage for persistent or worsening symptoms without radiological progression; (4) operation time and length of hospital stay; (5) postoperative complication rate; and (6) number of imaging follow-ups.

### 2.4 Imaging assessment and follow-up

All patients underwent a baseline non-contrast cranial CT scan within 24–48 h after surgery. Routine imaging follow-up was performed at 1 month and 6 months postoperatively, and thereafter every 6 months or whenever clinically indicated. Follow-up was conducted through outpatient visits and telephone interviews, with the censoring date set at August 1, 2026, or at the time of patient death.

To minimize detection bias, an independent senior radiologist (associate chief physician), who was blinded to group allocation and had no involvement in patient care, re-measured haematoma thickness and midline shift on all preoperative and follow-up cranial CT scans. Inter-rater agreement between the original and independent measurements was assessed using the intraclass correlation coefficient (ICC).

### 2.5 Statistical analysis

Statistical analyses were performed using SPSS 26.0 and R 4.2.1 software. Continuous variables were first tested for normality using the Shapiro–Wilk test. Normally distributed data were expressed as mean ± standard deviation, and comparisons among the three groups were made using one-way analysis of variance (ANOVA), followed by Tukey’s HSD test for pairwise comparisons. Non-normally distributed data were presented as median (interquartile range), and group comparisons were performed using the Kruskal–Wallis *H* test, with Dunn’s test for *post hoc* pairwise comparisons. Categorical variables were expressed as counts (percentages) and compared using the Pearson chi-square test. For outcomes with statistically significant overall differences, post-hoc pairwise comparisons were conducted with Bonferroni correction for multiple testing. All tests were two-sided, and a *p*-value <0.05 was considered statistically significant. Because of the small number of outcome events, multivariable adjustment and propensity score matching were not feasible; unadjusted analyses were therefore used. Secondary outcomes were exploratory, and no correction for multiple testing was applied.

Sample size calculation. Based on previous clinical observations, the 6-month recurrence rates were assumed to be no higher than 5% in the MMAE and NE-MMAD groups and approximately 20% in the BHD group. With a two-sided α of 0.05, a power of 0.80, and a group ratio of 1:1:3, the required total sample size was calculated to be 191 patients using PASS 2021. The present study included 265 patients, which met the statistical requirement. Because the number of recurrence events in the MMAE and NE-MMAD groups was small, 95% confidence intervals for the recurrence rates have been provided.

## 3 Results

### 3.1 Baseline characteristics

A total of 265 patients with CSDH were enrolled, including 46 (17.36%) in the MMAE group, 152 (57.36%) in the BHD group, and 67 (25.28%) in the NE-MMAD group. No significant differences were observed among the three groups in terms of age, sex, haematoma side, haematoma thickness, septated haematoma, midline shift, baseline mRS score, anticoagulant/antiplatelet therapy, hypertension, or diabetes mellitus (all *p* > 0.05), indicating that the baseline characteristics were well balanced across the groups (Table 1).

**Table 1:** Comparison of baseline demographic and clinical characteristics among the MMAE, BHD, and NE-MMAD groups.

Variable	Total (*n* = 265)	MMAE group (*n* = 46)	BHD group (*n* = 152)	NE-MMAD group (*n* = 67)	*p*-value
Age (years), median (IQR)	67.00 (60.00, 73.00)	68.00 (60.00, 74.00)	67.00 (59.50, 73.00)	67.00 (58.50, 73.00)	*χ*^2^ = 0.61^#^, *p* = 0.739
Sex, *n* (%)					*χ*^2^ = 0.71, *p* = 0.700
Male	196 (73.96)	32 (69.57)	115 (75.66)	49 (73.13)	
Female	69 (26.04)	14 (30.43)	37 (24.34)	18 (26.87)	
Haematoma side, *n* (%)					*χ*^2^ = 0.24, *p* = 0.993
Bilateral	74 (27.92)	12 (26.09)	44 (28.95)	18 (26.87)	
Right	93 (35.09)	16 (34.78)	53 (34.87)	24 (35.82)	
Left	98 (36.98)	18 (39.13)	55 (36.18)	25 (37.31)	
Haematoma thickness (mm), median (IQR)	22.00 (19.00, 25.00)	21.00 (18.00, 25.00)	22.00 (19.00, 25.00)	22.00 (19.00, 25.00)	*χ*^2^ = 0.95^#^, *p* = 0.622
Septated haematoma, *n* (%)	40 (15.09)	6 (13.04)	20 (13.16)	14 (20.90)	*χ*^2^ = 2.36, *p* = 0.307
Midline shift (mm), median (IQR)	7.00 (5.00, 8.00)	6.50 (5.00, 8.00)	7.00 (5.00, 8.00)	7.00 (5.00, 8.00)	*χ*^2^ = 0.38^#^, *p* = 0.828
Baseline mRS, *n* (%)					*χ*^2^ = 0.17, *p* = 0.920
>2	114 (43.02)	19 (41.30)	67 (44.08)	28 (41.79)	
≤2	151 (56.98)	27 (58.70)	85 (55.92)	39 (58.21)	
Anticoagulation/antiplatelet therapy, *n* (%)	100 (37.74)	16 (34.78)	59 (38.82)	25 (37.31)	*χ*^2^ = 0.25, *p* = 0.882
Hypertension, *n* (%)	167 (63.02)	29 (63.04)	95 (62.50)	43 (64.18)	*χ*^2^ = 0.06, *p* = 0.972
Diabetes mellitus, *n* (%)	91 (34.34)	14 (30.43)	55 (36.18)	22 (32.84)	*χ*^2^ = 0.61, *p* = 0.738

Data are *n* (%) or median (IQR). *χ*^2^, Pearson chi-square test; ^#^Kruskal–Wallis test. *p*-values are for overall comparisons among the three groups.

### 3.2 Primary outcome: recurrence rate

At 6 months postoperatively, recurrence occurred in 35 patients (13.21%) overall. The recurrence rates in the MMAE group (4.35%, 2/46) and the NE-MMAD group (4.48%, 3/67) were significantly lower than that in the BHD group (19.74%, 30/152; *χ*^2^ = 13.26, *p* = 0.001). No significant difference was observed between the MMAE and NE-MMAD groups (*p* > 0.05). Inter-rater agreement between the original and independent measurements was excellent for haematoma thickness (ICC = 0.941) and for midline shift (ICC = 0.927). The recurrence rates based on the independent measurements were consistent with the original analysis (MMAE: 4.35%; BHD: 19.74%; NE-MMAD: 4.48%; *p* = 0.001).

The 6-month recurrence rate was 4.35% (95% CI, 0.53–14.84%) in the MMAE group, 19.74% (95% CI, 13.73–26.96%) in the BHD group, and 4.48% (95% CI, 0.93–12.53%) in the NE-MMAD group. The odds of recurrence were significantly higher in the BHD group than in the MMAE group (OR = 5.41, 95% CI, 1.24–23.58) and the NE-MMAD group (OR = 5.25, 95% CI, 1.54–17.85), with no significant difference between the MMAE and NE-MMAD groups (OR = 1.03, 95% CI, 0.17–6.43).

### 3.3 Secondary outcomes

The NE-MMAD group had the highest rate of neurological improvement (88.06%, 59/67), which was significantly superior to that of the MMAE group (60.87%, 28/46) and the BHD group (71.71%, 109/152; *χ*^2^ = 11.41, *p* = 0.003). No significant difference was detected between the MMAE and BHD groups (*p* > 0.05). The neurological improvement rate was 60.87% (95% CI, 45.37–74.91%) in the MMAE group, 71.71% (95% CI, 63.84–78.71%) in the BHD group, and 88.06% (95% CI, 77.82–94.70%) in the NE-MMAD group. The odds of improvement were significantly higher in the NE-MMAD group than in the BHD group (OR = 0.34 for BHD vs. NE-MMAD, 95% CI, 0.15–0.78). The odds of improvement were also higher in the NE-MMAD group than in the MMAE group (OR = 4.74 for NE-MMAD vs. MMAE, 95% CI, 1.84–12.22), with no significant difference between the MMAE and BHD groups (OR = 1.63 for BHD vs. MMAE, 95% CI, 0.82–3.25).

The reoperation rates in the MMAE group (4.35%, 2/46) and the NE-MMAD group (4.48%, 3/67) were both significantly lower than that in the BHD group (19.08%, 29/152; *χ*^2^ = 12.45, *p* = 0.002), with no significant difference between the MMAE and NE-MMAD groups (*p* > 0.05). Among the 30 patients with recurrence in the BHD group, 29 underwent reoperation, and 1 did not undergo reoperation because symptoms were mild. The reoperation rate was 4.35% (95% CI, 0.53–14.84%) in the MMAE group, 19.08% (95% CI, 13.17–26.24%) in the BHD group, and 4.48% (95% CI, 0.93–12.53%) in the NE-MMAD group.

In addition, 5 patients (10.87%) in the MMAE group underwent salvage burr hole drainage during the same hospitalization due to persistent or worsening symptoms without radiological progression; these cases were not included in the reoperation rate calculation. The mean preoperative haematoma thickness in these 5 patients was 21.40 mm (range, 17–28 mm), and the mean midline shift was 7.00 mm (range, 4–10 mm).

When “any additional surgery” (reoperation for recurrence plus salvage burr hole drainage) was analyzed as a composite outcome, the rates were 15.2% (7/46) in the MMAE group, 19.1% (29/152) in the BHD group, and 4.5% (3/67) in the NE-MMAD group (*χ*^2^ = 7.91, *p* = 0.019). After Bonferroni correction for pairwise comparisons, the rate was significantly lower in the NE-MMAD group than in the BHD group (*p* = 0.005), whereas no significant differences were observed between the MMAE and BHD groups or between the MMAE and NE-MMAD groups. The rate of any additional surgery was 15.22% (95% CI, 6.34–28.87%) in the MMAE group, 19.08% (95% CI, 13.17–26.24%) in the BHD group, and 4.48% (95% CI, 0.93–12.53%) in the NE-MMAD group.

### 3.4 Perioperative indicators and safety

The operation time was shortest in the MMAE group (31.00 min) and longest in the NE-MMAD group (70.00 min; H = 192.53, *p* < 0.001). The hospital stay was shorter in the MMAE group (7.00 days) than in the BHD group (9.00 days; *p* = 0.030), whereas the NE-MMAD group (8.00 days) showed no significant difference compared with the other two groups. The number of imaging follow-ups was significantly lower in both the MMAE group (4.00) and the NE-MMAD group (4.00) than in the BHD group (6.00; *H* = 75.22, *p* < 0.001).

The complication rates in the three groups were 8.70% (4/46), 9.21% (14/152), and 5.97% (4/67), respectively, with no statistically significant difference (*χ*^2^ = 0.65, *p* = 0.722). Notably, one patient in the MMAE group developed permanent blindness due to inadvertent embolization through a dangerous meningo-ophthalmic anastomosis; no such severe complication occurred in the other two groups. The complication rate was 8.70% (95% CI, 2.42–20.79%) in the MMAE group, 9.21% (95% CI, 5.13–14.97%) in the BHD group, and 5.97% (95% CI, 1.65–14.59%) in the NE-MMAD group (Table 2 and Figure 4).

**Table 2:** Comparison of perioperative indicators and surgical outcomes among the MMAE, BHD, and NE-MMAD groups.

Variable	Total (*n* = 265)	MMAE group (*n* = 46)	BHD group (*n* = 152)	NE-MMAD group (*n* = 67)	*p*-value	OR (95% CI) BHD vs. MMAE	OR (95% CI) NE-MMAD vs. MMAE	OR (95% CI) BHD vs. NE-MMAD
6-month recurrence rate, *n* (%) [95% CI]	35 (13.21) [9.37–17.89]	2 (4.35) [0.53–14.84]a	30 (19.74) [13.73–26.96]b	3 (4.48) [0.93–12.53]a	*χ*^2^ = 13.26, *p* = 0.001	5.41 (1.24–23.58)	1.03 (0.17–6.43)	5.25 (1.54–17.85)
Neurological improvement rate, *n* (%) [95% CI]	196 (73.96) [68.24–79.14]	28 (60.87) [45.37–74.91]a	109 (71.71) [63.84–78.71]a	59 (88.06) [77.82–94.70]b	*χ*^2^ = 11.41, *p* = 0.003	1.63 (0.82–3.25)	4.74 (1.84–12.22)	0.34 (0.15–0.78)
Reoperation rate, *n* (%) [95% CI]	34 (12.83) [9.05–17.47]	2 (4.35) [0.53–14.84]a	29 (19.08) [13.17–26.24]b	3 (4.48) [0.93–12.53]a	*χ*^2^ = 12.45, *p* = 0.002	5.19 (1.19–22.64)	1.03 (0.17–6.43)	5.03 (1.48–17.15)
Any additional surgery, *n* (%) [95% CI]	39 (14.72) [10.68–19.57]	7 (15.22) [6.34–28.87]^ab^	29 (19.08) [13.17–26.24]^a^	3 (4.48) [0.93–12.53]^b^	*χ*^2^ = 7.91, *p* = 0.019	1.31 (0.53–3.23)	0.26 (0.06–1.07)	5.03 (1.48–17.15)
Complications, *n* (%) [95% CI]	22 (8.30) [5.28–12.30]	4 (8.70) [2.42–20.79]	14 (9.21) [5.13–14.97]	4 (5.97) [1.65–14.59]	*χ*^2^ = 0.65, *p* = 0.722	1.07 (0.33–3.41)	0.67 (0.16–2.81)	1.60 (0.51–5.05)
Operation time (minutes), median (IQR)	48.00 (40.00, 60.00)	31.00 (28.00, 38.00)^a^	45.00 (41.00, 50.00)^b^	70.00 (65.00, 76.50)^c^	*χ*^2^ = 192.53^#^, *p* < 0.001	–	–	–
Hospital stay (days), median (IQR)	8.00 (7.00, 11.00)	7.00 (6.00, 11.00)^a^	9.00 (7.00, 11.00)^b^	8.00 (7.00, 10.00)^ab^	*χ*^2^ = 7.00^#^, *p* = 0.030	–	–	–
Imaging follow-ups (*n*), median (IQR)	5.00 (4.00, 7.00)	4.00 (3.00, 5.00)^a^	6.00 (5.00, 8.00)^b^	4.00 (4.00, 5.00)^a^	*χ*^2^ = 75.22^#^, *p* < 0.001	–	–	–

Data are *n* (%) [95% CI] or median (IQR). Only event (Yes) is shown; No = total − Yes. ORs (95% CI) are shown where available; “–” = not applicable. *χ*^2^, Pearson chi-square test; ^#^Kruskal–Wallis test. Same superscript letter = no significant difference (*p* > 0.05). *p*-values are for overall comparisons among the three groups.

**Figure 4:**
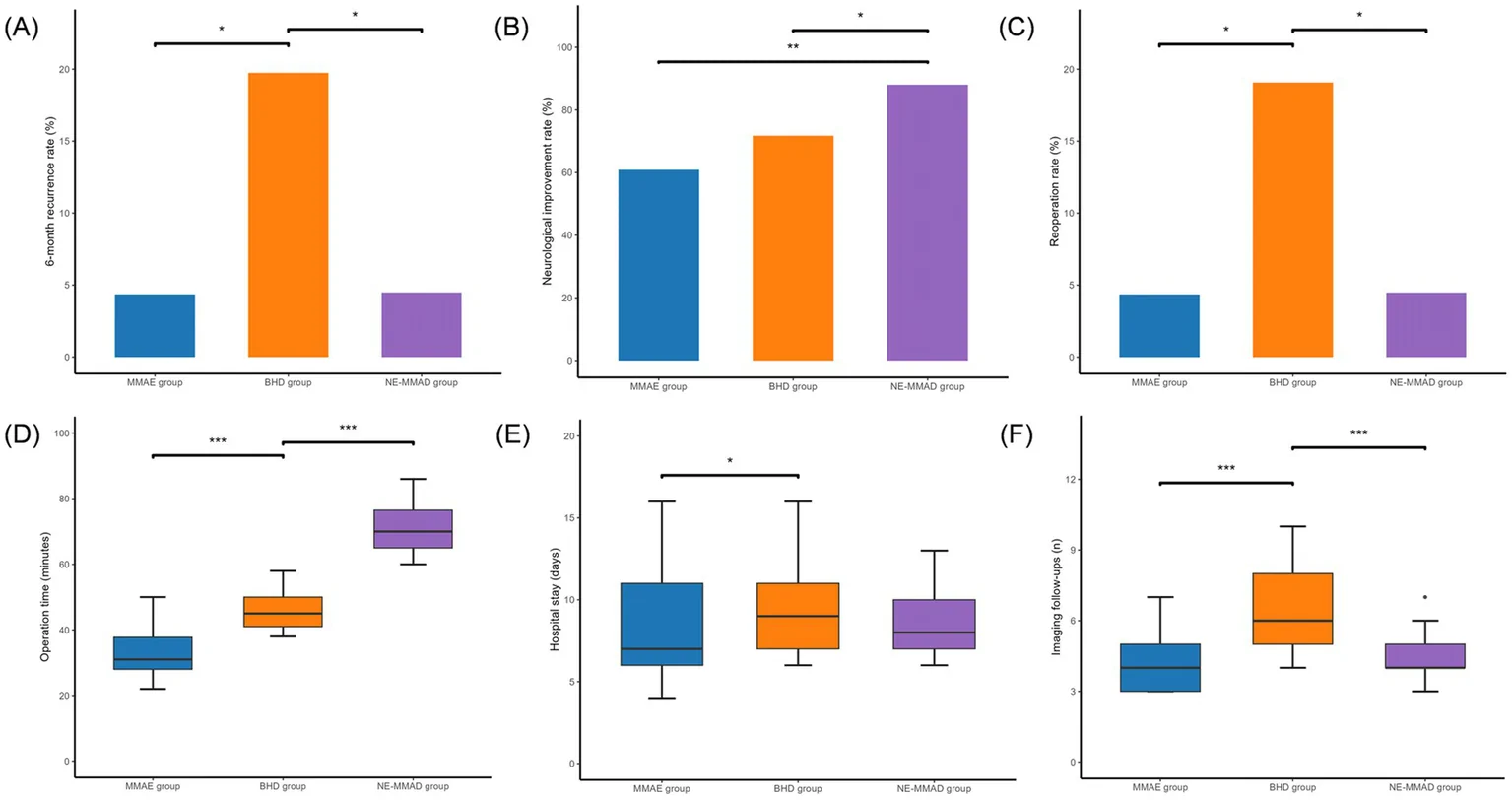
Comparison of clinical outcomes among the MMAE, BHD, and NE-MMAD groups. **(A)** Six-month recurrence rate; **(B)** neurological improvement rate; **(C)** reoperation rate; **(D)** operation time (minutes); **(E)** hospital stay (days); **(F)** imaging follow-ups (n). Bar graphs in **(A–C)** display percentages. Box-and-whisker plots in **(D–F)** display medians with interquartile ranges (boxes) and ranges (whiskers). Significance is indicated as follows: *p < 0.05, **p < 0.01, ***p < 0.001.

Complications graded by Clavien-Dindo are summarized in Supplementary Table S1. In the MMAE group, there were 1 Grade I (local subcutaneous haematoma), 1 Grade II (contrast allergy), 1 Grade IIIa (pseudoaneurysm), and 1 Grade IV (permanent blindness) event; in the BHD group, 7 Grade I, 3 Grade II, and 4 Grade IIIb events; and in the NE-MMAD group, 1 Grade I, 1 Grade II, and 2 Grade IIIb events.

## 4 Discussion

### 4.1 Pathophysiological mechanisms of CSDH and the theoretical basis of the three surgical modalities

This multicenter retrospective analysis compared the efficacy and safety of MMAE, BHD, and NE-MMAD for CSDH. The significant differences among the three groups in recurrence control and neurological improvement should be interpreted from the perspective of CSDH pathophysiology. Current evidence indicates that CSDH originates from an aseptic inflammatory response of dural border cells, which subsequently activates macrophage infiltration and the release of proinflammatory cytokines such as TNF-α, IL-6, and VEGF, ultimately forming a self-perpetuating pathological cycle of “inflammation-angiogenesis-microhaemorrhage” driven by recurrent neovascular microbleeding (17). Based on this mechanistic understanding, the treatment paradigm has evolved from simple “haematoma evacuation” to a comprehensive model combining “evacuation with etiological intervention.”

The three surgical modalities differ in their pathophysiological targets: BHD merely removes the accumulated haematoma fluid without directly addressing the source of persistent neovascular leakage, thus yielding the highest recurrence rate (18). MMAE occludes the MMA trunk via an endovascular approach, interrupting the nutritional supply to the neovessels of the outer membrane at its source, but it cannot immediately eliminate the established haematoma mass (19). In contrast, NE-MMAD achieves thorough haematoma clearance and removal of inflammatory stimuli under direct visualization, while simultaneously physically disconnecting the frontal trunk and its branches of the MMA (20), thereby accomplishing the dual goals of “relieving mass effect” and “eliminating the underlying cause.” These mechanistic differences provide a reasonable explanation for the disparities in recurrence rates and neurological improvement observed among the three groups in this study.

### 4.2 Comprehensive advantages and clinical positioning of NE-MMAD

Among the three modalities, the NE-MMAD group was associated with the most favorable outcomes in terms of recurrence rate and neurological improvement compared with the other two groups. This advantage is attributable to the triple goals simultaneously accomplished by NE-MMAD: resetting the inflammatory microenvironment, interrupting the causative blood supply, and achieving early brain decompression.

First, thorough elimination of the inflammatory microenvironment. The pathological essence of CSDH has been redefined as a localized inflammatory disease characterized by sustained inflammation, pathological angiogenesis, and hyperfibrinolysis (21, 22). The outer membrane and septations of the haematoma are rich in proinflammatory factors such as VEGF, IL-6, and TNF-α, which constitute an inflammatory microenvironment driving neovascular leakage and haematoma expansion (23). Studies have demonstrated that levels of inflammatory markers such as IL-6 and IL-8 in haematoma fluid are significantly correlated with poorer neurological outcomes and greater haematoma burden, and that higher IL-6 concentrations in haematoma fluid, along with stronger VEGF and bFGF expression in the outer membrane, are associated with an increased risk of recurrence (24, 25).

Against this pathophysiological backdrop, the three modalities exert fundamentally different effects on the inflammatory microenvironment. BHD can drain the liquid component of the haematoma, but under blind operation, it cannot thoroughly remove solid clots and fibrous septations deposited in various recesses of the cavity; these residuals become a “reservoir” for sustained release of inflammatory mediators. MMAE, by blocking MMA blood supply, induces ischemic changes in the outer membrane and alleviates inflammatory congestion, thereby indirectly suppressing the ongoing production of inflammatory cytokines; however, the inflammatory mediators and old clots already accumulated within the cavity are not removed and must rely on slow spontaneous resorption. In contrast, NE-MMAD, under high-definition endoscopic visualization, systematically evacuates liquid haematoma, solid clots, and fibrous septations (26), physically removing the inflammatory-factor-rich content in a single session, effectively achieving a “chemical reset” of the local inflammatory microenvironment by markedly reducing the concentrations of proinflammatory mediators such as VEGF and IL-6, thereby breaking the vicious cycle of “inflammation-angiogenesis-microhaemorrhage-reinflammation.”

Second, definitive disconnection of MMA blood supply. Based on precise preoperative localization using three-dimensional reconstruction, the frontal trunk and branches of the MMA are thoroughly disconnected under direct vision using bipolar coagulation (27). Compared with MMAE, NE-MMAD provides more complete vascular disconnection—it can directly address small perforating branches without the risk of reflux or inadvertent embolization. Compared with BHD, the definitive disconnection of MMA blood supply fundamentally eliminates the source of persistent leakage from neovessels in the outer membrane. The physical clearance of the inflammatory microenvironment and the physical disconnection of MMA blood supply exert a synergistic effect: removing existing inflammatory stimuli while blocking potential future inflammatory blood supply. The fundamental reason for the significantly lower recurrence rate with NE-MMAD compared with BHD lies in this dual mechanism of “clearance plus blockade.”

Third, early brain decompression promotes neurological recovery. Unlike MMAE, which requires weeks to months for slow haematoma resorption (Figure 1E), NE-MMAD achieves a marked reduction in haematoma volume and significant relief of mass effect as early as postoperative day 1 (Figure 3H). Early brain decompression creates the most favorable conditions for cerebral perfusion restoration, venous return improvement, and axonal remodeling (28, 29). In this study, the neurological improvement rate in the NE-MMAD group was higher than in the other two groups, suggesting that the timeliness of mass effect relief, combined with the thoroughness of inflammatory microenvironment resetting, may contribute to neurological prognosis (30).

Based on the above evidence, NE-MMAD should be prioritized for the following CSDH subtypes: (1) septated, multiloculated, or organized haematomas as indicated by imaging (preoperative MRI or contrast-enhanced CT can clearly delineate septations and internal architecture); (2) recurrent CSDH; and (3) cases with significant mass effect in patients whose general condition permits general anesthesia. For patients meeting these indications, NE-MMAD offers the most substantial comprehensive benefits in terms of recurrence control and functional recovery.

### 4.3 Indications, advantages, and risk boundaries of MMAE

In this cohort, MMAE was associated with a significantly lower recurrence rate than BHD, with the shortest operation time and hospital stay among the three groups, fully demonstrating its minimally invasive and efficient profile. However, this study revealed two limitations that warrant high vigilance.

First, delayed resolution of mass effect and the necessity of salvage drainage. After MMAE interrupts MMA blood supply, complete haematoma resorption takes approximately 89 days (31). During this “resorption window,” the inflammatory exudates and old clots within the haematoma cavity remain uncleared, and with reduced compliance of the aging brain and slow recovery of compressed venous return, the brain tissue with significant mass effect cannot rapidly reposition. The literature reports that the proportion of patients requiring surgical salvage after MMAE alone is approximately 10–15% (32, 33). In our cohort, 5 patients (10.87%) underwent salvage burr hole drainage due to persistent symptoms postoperatively. Their mean preoperative haematoma thickness was 21.40 mm and mean midline shift was 7.00 mm—values close to or exceeding the median levels of the MMAE group (thickness 21 mm, midline shift 6.5 mm). These observations are based on a small number of cases (*n* = 5) and should therefore be regarded as hypothesis-generating rather than conclusive. Whether patients with more severe mass effect are at higher risk of requiring salvage drainage after MMAE alone requires confirmation in larger prospective studies. Previous studies have similarly reported that increased haematoma thickness and midline shift in CSDH are significantly associated with a higher risk of secondary intervention after standalone embolization (19, 34). These observations suggest that, for CSDH with significant mass effect, combined burr hole drainage or direct NE-MMAD may be considered; however, this requires confirmation in larger prospective studies. MMAE is more suitable as a prophylactic measure against recurrence rather than as an emergency intervention for rapid mass effect relief (35, 36).

It is worth emphasizing that the above 5 cases of salvage drainage are not equivalent to “reoperation for recurrence”—in these patients, imaging showed no haematoma enlargement, and the suboptimal symptom improvement was attributable to slow haematoma resorption and delayed resolution of mass effect, rather than true recurrence. After salvage drainage, all 5 patients experienced rapid symptom resolution and none had recurrence during long-term follow-up, further supporting the efficacy of MMAE in etiological interruption and suggesting that its combination with BHD may be a reasonable strategy for cases with significant mass effect.

To further address the issue of outcome definition, we added a composite outcome of “any additional surgery,” which includes reoperation for recurrence and salvage burr hole drainage. As reported in the Results and Table 2, the NE-MMAD group had the lowest rate of any additional surgery among the three groups. This composite outcome allows readers to more comprehensively assess the true intervention burden of MMAE, rather than focusing solely on the recurrence rate.

Second, the risk of blindness from dangerous meningo-ophthalmic anastomosis. In this group, one patient developed permanent blindness due to inadvertent embolization through a dangerous anastomosis (37). Dangerous anastomoses between the MMA and the ophthalmic artery can occur via orbital and lacrimal branches, with a reported incidence of approximately 0.5% (38); once embolic material refluxes into the ophthalmic artery and occludes the central retinal artery, the consequence is permanent blindness. Therefore, preoperative high-resolution MRA or DSA is mandatory to meticulously assess the anatomical relationship between the MMA and the ophthalmic artery, and intraoperative manipulation should be performed cautiously under multi-angle DSA roadmap guidance. If a dangerous anastomosis or high-risk reflux is identified, embolization should be immediately aborted and NE-MMAD considered instead.

Despite these limitations, MMAE retains irreplaceable value in the following scenarios: (1) elderly or medically frail patients who cannot tolerate general anesthesia; (2) patients on long-term anticoagulant/antiplatelet therapy who cannot safely discontinue these agents; (3) bilateral CSDH or prophylactic treatment for recurrence after BHD; and (4) patients in whom interventional facilities are adequate and no dangerous anastomosis is detected on evaluation. In these populations, the minimally invasive benefits of MMAE substantially outweigh its risks.

### 4.4 Clinical value and applicable boundaries of BHD

Although BHD had significantly higher recurrence and reoperation rates than MMAE and NE-MMAD in this study, it remains an irreplaceable foundational procedure in the CSDH treatment armamentarium. The core advantages of BHD include: (1) simplicity of operation, feasible under local anesthesia, particularly suitable for emergency settings; (2) low equipment requirements, without dependence on DSA or neuroendoscopy, enabling widespread application in primary care hospitals; (3) the lowest medical cost, with no expensive consumables; and (4) immediate relief of mass effect, with midline shift reversal observed as early as postoperative day 1 (Figure 2F).

In our BHD group, 122 of 152 patients (80.26%) remained recurrence-free at 6 months postoperatively. For patients with first-onset CSDH showing homogeneous haematoma without obvious septations on imaging, BHD, with its simplest procedure and lowest cost, should remain the first-line choice (39).

Furthermore, BHD plays an important role in combination strategies—in this study, the MMAE patients who underwent salvage BHD all achieved symptom relief without further recurrence, and the postoperative drainage tube placement in the NE-MMAD group also falls within the scope of BHD. BHD remains the standard treatment for CSDH and serves as a fundamental component of combination strategies (40, 41).

### 4.5 Limitations

This study has several limitations. First, its retrospective design inevitably introduces selection bias and residual confounding, as treatment allocation was influenced by operator preference, patient willingness, and available resources. Although septation was assessed and baseline characteristics were balanced, unmeasured factors such as frailty, disease severity, and operator experience may still confound the results, and the analyses were unadjusted. Second, the sample size was relatively small, particularly in the MMAE (*n* = 46) and NE-MMAD (*n* = 67) groups, and the number of recurrence events was low. This precluded multivariable adjustment, propensity score matching, and subgroup analyses, and also prevented reliable time-to-event analysis because the exact timing of recurrence could not be accurately determined or was frequently missing; the binary recurrence outcome may therefore lose temporal information. Third, secondary outcomes were exploratory and no correction for multiple testing was applied; significant secondary findings should therefore be interpreted with caution and may be subject to false-positive error. Fourth, follow-up was relatively short, and long-term recurrence (>1 year) requires further observation. Fifth, the number of imaging follow-ups differed among groups, reflecting the need for more frequent CT monitoring in patients with drainage tubes; although all patients completed 6-month follow-up and recurrence was defined radiologically, surveillance bias cannot be entirely excluded. Sixth, although CT thickness was independently re-assessed by a blinded radiologist, mRS scores were recorded by the treating team and could not be independently blinded; mRS was partly assessed by telephone or video and was analyzed as a binary outcome, which may reduce sensitivity to smaller functional changes and introduce observer bias. However, the primary endpoint (recurrence) was based on blinded radiological assessment. Seventh, reoperation thresholds may differ across centers, and the reoperation rate should be interpreted with caution, although the primary radiological endpoint is not affected. Eighth, the study spanned more than 5 years, and center and time effects could not be adjusted because of the small number of events. Ninth, no systematic health economic evaluation was performed. Finally, although only one case of blindness occurred in the MMAE group, its clinical implications are substantial and have been discussed separately.

Future prospective, multicenter, large-sample randomized controlled trials are warranted to conduct more rigorous direct comparisons among the different surgical modalities, and machine learning approaches may be employed to develop individualized surgical recommendation models based on patient-specific characteristics, thereby providing higher-level evidence for personalized CSDH management.

## 5 Conclusion

In this cohort, MMAE and NE-MMAD were associated with lower recurrence and reoperation rates compared with BHD, and NE-MMAD was associated with the greatest neurological improvement. MMAE offers the advantages of minimal invasiveness and rapid recovery, but carries the risks of blindness and delayed resolution of mass effect; it should not be the first-line choice for patients with significant mass effect. NE-MMAD may be preferred for septated, organized, recurrent, and markedly space-occupying haematomas. Although BHD was associated with a relatively higher recurrence rate, its simplicity, low cost, and wide accessibility make it a reasonable first-line option for first-onset, non-septated CSDH. Clinical decision-making should integrate haematoma characteristics, patient status, and available medical resources to guide individualized stratified treatment.

## Data Availability

The raw data supporting the conclusions of this article will be made available by the authors, without undue reservation.
