# Supplementary material for: Comparative outcomes of middle meningeal artery embolization, burr hole drainage, and neuroendoscopic-assisted evacuation with MMA disconnection for chronic subdural haematoma: a multicenter retrospective cohort study
Source: Front Neurol. 2026 Oct 19;17:1975364. doi: 10.3389/fneur.2026.1975364 (PMC13724487; doi:10.3389/fneur.2026.1975364)
Supplement: Supplementary file 1 [file Table_1.docx]

Supplementary Table S1. Clavien-Dindo classification of postoperative complications among the three groups

| **Clavien-Dindo Grade** | **MMAE group (n = 46)** | **BHD group (n = 152)** | **NE-MMAD group (n = 67)** | **Specific complications** |
| --- | --- | --- | --- | --- |
| Grade I | 1 | 7 | 1 | MMAE: local subcutaneous haematoma (n = 1); BHD: subdural effusion (n = 7); NE-MMAD: transient neurological deterioration (n = 1) |
| Grade II | 1 | 3 | 1 | MMAE: contrast allergy (n = 1); BHD: seizures (n = 2), intracranial infection (n = 1); NE-MMAD: wound infection (n = 1) |
| Grade IIIa | 1 | 0 | 0 | MMAE: pseudoaneurysm at puncture site (n = 1) |
| Grade IIIb | 0 | 4 | 2 | BHD: tension pneumocephalus (n = 3), subdural haematoma (n = 1); NE-MMAD: subdural haematoma (n = 2) |
| Grade IV | 1 | 0 | 0 | MMAE: permanent blindness (n = 1) |
| Grade V | 0 | 0 | 0 | — |
| Total | 4 | 14 | 4 |  |

**Note:** Grade IV in the MMAE group refers to one patient who developed permanent blindness due to inadvertent embolization through a dangerous meningo-ophthalmic anastomosis. This catastrophic adverse event is discussed separately in the Discussion section of the main manuscript.
